# DCE-MRI Background Parenchymal Enhancement Quantified from an Early versus Delayed Post-contrast Sequence: Association with Breast Cancer Presence

**DOI:** 10.1038/s41598-017-02341-8

**Published:** 2017-05-18

**Authors:** Shandong Wu, Margarita L. Zuley, Wendie A. Berg, Brenda F. Kurland, Rachel C. Jankowitz, Jules H. Sumkin, David Gur

**Affiliations:** 1 Departments of Radiology, Biomedical Informatics, and Bioengineering, University of Pittsburgh, 4200 Fifth Ave, Pittsburgh, PA 15260 USA; 2 Magee-Womens Hospital of University of Pittsburgh Medical Center, 300 Halket St, Pittsburgh, PA 15213 USA; 3 University of Pittsburgh Cancer Institute, Department of Biostatistics, University of Pittsburgh, 4200 Fifth Ave, Pittsburgh, PA 15260 USA; 4 Department of Medicine, University of Pittsburgh, 4200 Fifth Ave, Pittsburgh, PA 15260 USA

## Abstract

We investigated automated quantitative measures of background parenchymal enhancement (BPE) derived from an early versus delayed post-contrast sequence in breast dynamic contrast-enhanced magnetic resonance imaging (DCE-MRI) for association with breast cancer presence in a case-control study. DCE-MRIs were retrospectively analyzed for 51 cancer cases and 51 controls with biopsy-proven benign lesions, matched by age and year-of-MRI. BPE was quantified using fully-automated validated computer algorithms, separately from three sequential DCE-MRI post-contrast-subtracted sequences (SUB1, SUB2, and SUB3). The association of BPE computed from the three SUBs and other known factors with breast cancer were assessed in terms of odds ratio (OR) and area under the receiver operating characteristic curve (AUC). The OR of breast cancer for the percentage BPE measure (BPE%) quantified from SUB1 was 3.5 (95% Confidence Interval: 1.3, 9.8; p = 0.015) for 20% increments. Slightly lower and statistically significant ORs were also obtained for BPE quantified from SUB2 and SUB3. There was no significant difference (p > 0.2) in AUC for BPE quantified from the three post-contrast sequences and their combination. Our study showed that quantitative measures of BPE are associated with breast cancer presence and the association was similar across three breast DCE-MRI post-contrast sequences.

## Introduction

Digital mammography is the standard clinical screening imaging modality for breast cancer. The masking effect for women with dense breasts reduces the sensitivity of mammography^1^. According to the American Cancer Society, a more sensitive modality, breast magnetic resonance imaging (MRI), is recommended as a supplementary examination to digital mammography^2^, for women considered at elevated risk of developing breast cancer, including typically women with known pathogenic *BReast CAncer susceptibility gene 1/2* (*BRCA1/2*) mutation(s) and their untested first-degree relatives as well as those with a 20% to 25% lifetime risk or higher as calculated by risk models which account for age of affected relatives. Annual surveillance with breast MRI has been shown to reduce incidence of advanced-stage breast cancer in women with pathogenic *BRCA1/2* mutations^3^.

Breast dynamic contrast-enhanced MRI (DCE-MRI) characterizes 3D anatomic properties of fibroglandular (i.e., dense) tissue (FGT) and the use of a contrast agent provides a sensitive way to characterize *in-vivo* physiologic and biologic activities in breast tissue^4^ that are relevant to breast cancer risk^5^. Contrast enhancement of normal breast tissue (other than breast tumors) in DCE-MRI is known as background parenchymal enhancement (BPE), which has been reported to be associated with breast cancer risk^6–8^, when visually assessed by the four qualitative Breast Imaging-Reporting and Data System (BI-RADS) categories (i.e., *minimal*, *mild*, *moderate*, or *marked*)^9^. Although clinically useful, BI-RADS-based BPE assessment is subjective, with high intra- and inter-reader variability^10, 11^, and there is particular difficulty distinguishing the categories of *mild* and *moderate*. Fully-automated computerized quantitative methods for objective and reproducible measurement of MRI BPE is in great clinical need.

In current clinical practice, screening breast MRI uses the same protocols as diagnostic breast MRI, including a pre-contrast and several (i.e., 3) post-contrast sequences acquired sequentially after injection of contrast agent^9, 12, 13^. Recently, abbreviated breast MRI protocols are emerging for breast cancer detection and lesion characterization^14–16^. In screening context the abbreviated MRI aims at developing a quicker and more cost-effective screening protocol^16^, by reducing certain MR sequences in current full DCE-MRI protocols^17^. The characteristics of BPE computed from the multiple post-contrast sequences in breast DCE-MRI merit further investigation. The purpose of this study was to investigate automated quantitative measures of BPE derived from an early versus delayed post-contrast sequence in breast DCE-MRI for association with breast cancer presence in a case-control study.

## Methods

### Dataset and imaging protocols

This retrospective study was Health Insurance Portability and Accountability Act (HIPAA) compliant and received Institutional Review Board (IRB) approval by the University of Pittsburgh, Human Research Protection Office (HRPO). Informed consent from patients was waived due to the retrospective nature of this study. All experiments and analyses were conducted in accordance with the Good Clinical Practice guidelines and regulations. The study cohort has been described in detail elsewhere^18^. To recap very briefly, we studied 102 women in a case-control setting, including 51 unilateral breast cancer cases (gold standard: pathology test) and 51 matched controls (by age [±3 years] and year-of-MRI [±1 year]) who had a single unilateral biopsy-proven benign lesion. Each participant had a suspicious unilateral abnormality rated as BI-RADS 4 or 5 in a diagnostic setting by digital mammography, ultrasound, and/or clinical exam from January 2009 to December 2011 at our institution; they consented to undergo bilateral breast MRI prior to percutaneous core and/or surgical biopsy. The cancer cases were not known to have cancer until after the MRI and pathology confirmation. No breast cancer was diagnosed for controls during an average of 3.7 years of follow up (range 1.4–5.5 years) since their MRI acquisitions.

Following the standard clinical breast MRI protocol at our institution, all DCE-MRIs were acquired by a 1.5T scanner (GE Signa EXCITE, GE Health, Nutley, NJ) in bilateral axial view, using a dedicated 7-channel surface array breast coil (InVivo, Gainesville, FL, USA). The DCE sequences were fat-suppressed and included a pre-contrast and three post-contrast sequences. Bolus injection of the contrast agent, ProHance (Bracco Diagnostics, Princeton, NJ), at 0.1 mmol/kg, 3 cc/sec was followed by a 20 cc saline flush. The first post-contrast sequence acquisition was centered at 90 seconds after contrast material injection. The temporal resolution of the post-contrast sequence acquisition was about 3 minutes for each sequence. Three corresponding subtraction sequences (i.e., SUB1, SUB2, and SUB3) were generated by subtracting the pre-contrast from each of the three post-contrast sequences, respectively. Major imaging parameters were: matrix 512 × 512; field of view 28–34 cm, slice thickness 2 mm; flip angle 10°, repetition time (TR) 5.68 msec, echo time (TE) 2.736 msec. The slice number of the bilateral axial scan ranged from 68 to 160 depending on the size of the breasts.

A total of 153 breasts (51 unilaterally cancer-free breasts from the cancer cases and 102 breasts bilaterally from the controls) were studied for two breast-wise analyses. The main analysis (Comparison A) was to compare MRI BPE measures from the contralateral breasts of both cancer cases and controls. A robustness analysis (Comparison B) compared BPE from the biopsy-proven benign breast of controls to the contralateral breasts of cancer cases. Note that the ipsilateral side of the cancer cases was excluded from BPE analysis at this stage, as our current BPE quantification algorithm has not yet been validated in processing cancer-affected breasts.

### MRI BPE quantification

**Figure 1:**
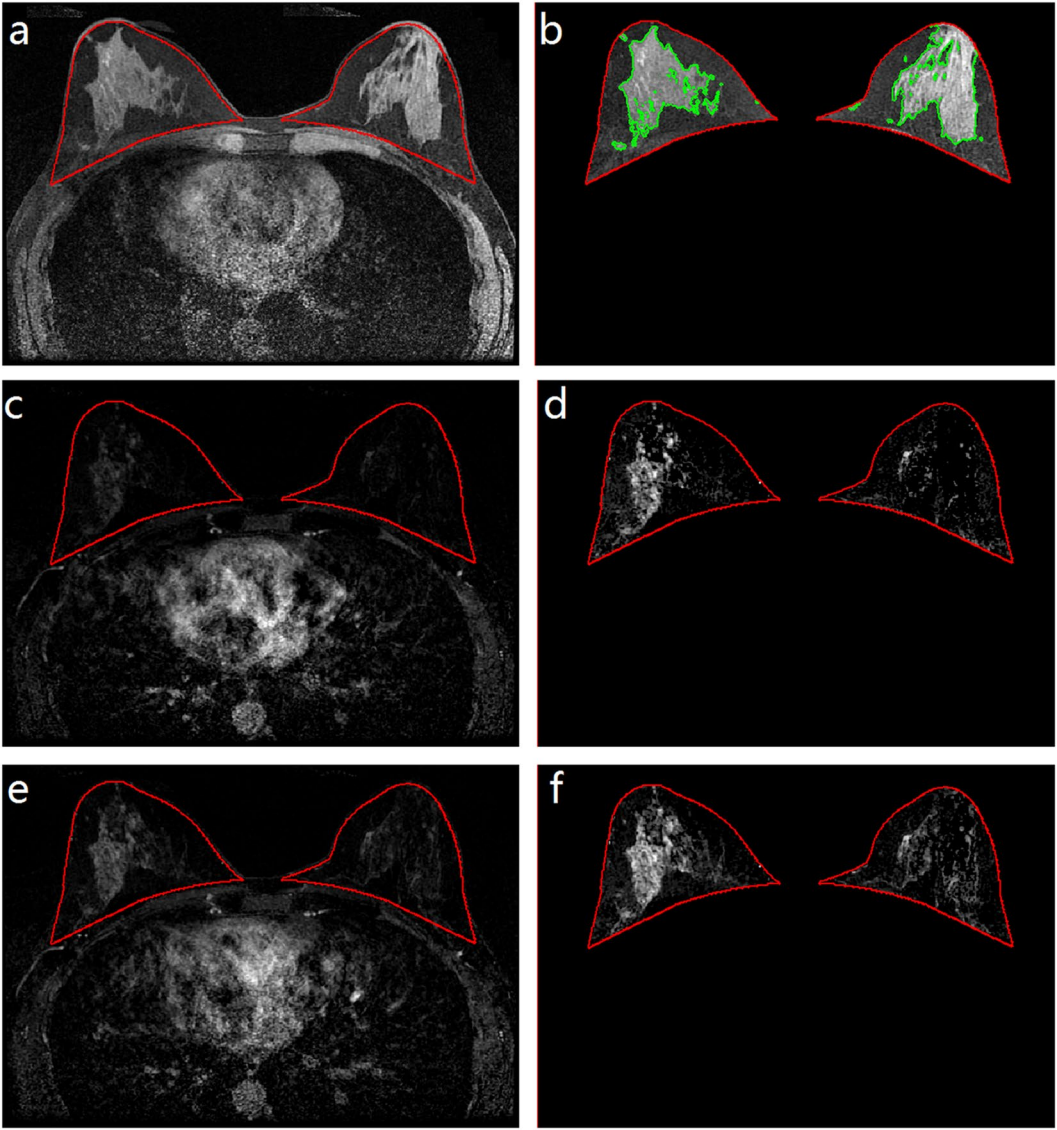
Automated segmentation of background parenchymal enhancement (BPE) and fibroglandular tissue (FGT) from breast DCE-MRI scans. (**a**) Whole-breast segmentation (red contour) from a pre-contrast image. (**b**) FGT (outlined by green contour) estimated from the segmented whole-breast. (**c**) The corresponding first post-contrast-subtracted (SUB1) image with superimposed breast contour (red). (**d**) BPE estimated from the SUB1 image (plot **c**). (**e**) The corresponding third post-contrast-subtracted (SUB3) image with superimposed breast contour (red). (**f**) BPE estimated from the SUB3 image (plot **e**).

Previously published fully-automated computer algorithms^19–21^ were adapted (Fig. 1) to process breast DCE-MRI scans. Breast-wise BPE measures were computed separately from each of the three SUB sequences (the SUBs were aligned by rigid registration), with the goal of comparing the effect of BPE estimated from an early versus delayed post-contrast sequence. For breasts with benign findings, the benign lesion was included as part of “normal” tissue (as compared to cancerous tissue) in quantifying BPE for the robustness analysis. First, the breast region was separated from the other body parts (e.g., chest cavity) included in the breast MR images^19^ and the absolute total volume of the breast was computed (|Breast| in cm^3^). BPE was quantified within the breast region. The extent of MRI contrast enhancement was measured by a voxel-wise intensity enhancement ratio^20^:

$${\rm{R}} \% =({{\rm{I}}}_{{\rm{post}}}-{{\rm{I}}}_{{\rm{pre}}})/{{\rm{I}}}_{{\rm{pre}}}\times 100={{\rm{I}}}_{{\rm{sub}}}/{{\rm{I}}}_{{\rm{pre}}}\,\times 100,$$

where I denotes the corresponding voxel intensity value in the pre-contrast, post-contrast, and subtraction images. A breast-wise BPE measure (|BPE|) was summarized as the absolute total volume (cm^3^) of enhancing voxels whose enhancement ratios (R%) had an equal or greater value than a predefined cutoff threshold, R%_cutoff_. Here, an R%_cutoff_ value of 20% was selected referring to a previous risk-reducing intervention study performed on an independent breast MRI dataset (100 MRI scans)^20^; as a robustness analysis, BPE was also quantified by using R%_cutoff_ = 30% and R%_cutoff_ = 40%. In addition, a percentage-based breast-wise BPE measure was computed as $\mathrm{BPE} \% =|{\rm{BPE}}|\,/\,|{\rm{Breast}}|\times 100.$ Because of the nature of full automation, our automated BPE quantification algorithm is reproducible, generating exactly the same results for a given MRI scan.

### MRI FGT quantification and mammographic density estimation

For comparison purposes, breast density (fibroglandular tissue) measures were obtained. MRI FGT contents were segmented from the pre-contrast sequence of the MRI scan using a published fully-automated method^21^ (Fig. 1), generating two quantitative FGT measures: absolute total volume (|FGT|; unit: cm^3^) and relative percentage $\,\mathrm{FGT} \% =|{\rm{FGT}}|/\,|{\rm{Breast}}|\times 100$. Standard clinical assessment of mammographic density by BI-RADS density categories was retrieved from mammography reports of the digital mammograms acquired within 6 months prior to the analyzed MRI scans.

### Statistical analysis

First, we measured the correlations between the imaging variables of mammographic density, FGT, and BPE using Spearman’s rank-order correlation coefficient. Second, we computed odds ratios of the BPE measures computed from different SUBs with breast cancer using univariate and multivariable conditional logistic regression, where the multivariable regression controlled for four covariates: menopausal status (pre or post), family history of breast cancer, ordinal mammographic density, and quantitative FGT. Family history of breast cancer was encoded as binary (positive if at least one first, second, or third-degree family member was diagnosed with breast cancer). A family-wise approach, i.e., false discovery rate (FDR)^22^, was applied to adjust for multiple tests at the 0.05 FDR level. Third, we assessed the predictive ability of BPE for distinguishing the cancer cases from controls using unconditional logistic regression analysis and area under the receiver operating characteristic curve (AUC), and DeLong’s test for comparing the AUCs computed from different SUBs. Last, we showed preliminary effects of BPE over four known risk factors (i.e., age, menopausal status, family history, and ordinal mammographic density) in predicting the cancers from controls, where the likelihood ratio test was used to measure the statistical significance on the difference of the AUCs in nested models. All statistical tests were two-sided (Wald tests for conditional logistic regression), with p < 0.05 considered statistically significant. Statistical analyses were performed using SAS software (version 9.3 SAS Institute, Cary, NC).

## Results

### Patient and imaging characteristics

Table 1 shows detailed patient characteristics. Mammographic density and MRI measures are summarized in Table 2, separately for cases and controls. Mammographic density differed by >1 category only in one matched pair (scattered fibroglandular density for case, extremely dense for control). For amount of fibroglandular tissue, mammographic density and MRI FGT% were strongly correlated (Spearman’s correlation coefficient [SCC] was 0.60, p < 0.0001). At the same time, mammographic density and BPE% (from SUB1) were not correlated (SCC = −0.003, p = 0.97), indicating that BPE% may convey additional risk information as compared with the established risk factor of mammographic breast density. Comparing MRI measures of FGT and BPE, the absolute volume measures |FGT| and |BPE| were moderately correlated with SCC = 0.45 (SUB1), 0.50 (SUB2), and 0.50 (SUB3), respectively, all p < 0.0001, while the percentage measures FGT% and BPE% were not correlated with SCC = −0.03 (SUB1), 0.03 (SUB2), and 0.02 (SUB3), respectively, all p > 0.5. In terms of the correlations between bilateral breasts of a woman, MRI measures between the two breasts (benign lesion and contralateral) of controls showed a strong correlation, with SCC = 0.56 for BPE% (SUB 1) and SCC = 0.86 for FGT%, both p < 0.0001.

**Table 1:** Patient characteristics of the 102 patients including 51 breast cancer cases and 51 matched controls.

Characteristics		Cancer cases (n = 51) N (%)	Controls (n = 51) N (%)
Age (years): mean ± SD (range)		47.6 ± 7.4 (34–60)	47.1 ± 7.3 (31–60)
Menopausal status			
Premenopausal		28 (55%)	30 (59%)
Postmenopausal		23 (45%)	21 (41%)
MRI performed outside the second week of the menstrual cycle for premenopausal women		15 (29%)	15 (29%)
Diagnostic BI-RADS findings of single-side breast on mammography and/or ultrasound			
Breast with lesion (cancer/benign)	BI-RADS 4	11 (22%)	47 (92%)
	BI-RADS 5	40 (78%)	4 (8%)
Contralateral breast	BI-RADS 1	27 (53%)	21 (41%)
	BI-RADS 2	19 (37%)	25 (49%)
	BI-RADS 3	1 (2%)	1 (2%)
	BI-RADS 4	4 (8%)	4 (8%)
History of prior breast cancer		0 (0%)	0 (0%)
Family history of breast cancer		26 (51%)	31 (61%)
Family history of ovarian cancer		3 (6%)	0 (0%)
Known pathogenic *BRCA1/2* mutation		2 (4%)	0 (0%)
Prior biopsy (>1 year prior to the studied biopsy)			
Atypia		1 (2%)	0 (0%)
Benign abnormality		9 (18%)	6 (12%)
Exogenous hormone use			
Hormone replacement therapy		7 (14%)	5 (10%)
Birth control pills		33 (65%)	34 (67%)
Tamoxifen		1 (2%)	0 (0%)
None		5 (10%)	12 (24%)
Cancer type			
Ductal carcinoma *in situ* (DCIS)		2 (4%)	—
Invasive ductal carcinoma (IDC)		26 (51%)	—
Invasive lobular carcinoma (ILC)		4 (8%)	—
Mixed of IDC and DCIS		18 (35%)	—
Invasive mixed ductal-lobular carcinoma		1 (2%)	—
Tumor size			
≤2 cm		18 (35%)	—
2–5 cm		31 (61%)	—
>5 cm		2 (4%)	—

Data are numbers of subjects, with percentages in parentheses.

**Table 2:** Imaging measure characteristics in the contralateral breast for 51 breast cancer cases and 51 controls matched by age and year-of-MRI scan.

Imaging metrics	Cancer cases (n = 51)	Controls (n = 51)	P-value
Mammographic density (visual BI-RADS density categories): # of subjects (%)			0.45
Fatty	2 (4%)	1 (2%)	—
Scattered fibroglandular density	14 (27%	13 (25%)	—
Heterogeneously dense	32 (63%)	33 (65%)	—
Extremely dense	3 (6%)	4 (8%)	—
|FGT| (unit cm^3^): mean ± SD (range)	126 ± 88 (38–442)	117 ± 79 (24–524)	0.58
FGT% (%): mean ± SD (range)	13 ± 8 (5–40)	14 ± 7 (4–36)	0.59
|BPE| (unit cm^3^): mean ± SD (range)			
SUB1	494 ± 333 (135–1814)	358 ± 183 (84–1004)	0.014
SUB2	639 ± 415 (149–2173)	466 ± 246 (95–1161)	0.014
SUB3	685 ± 436 (168–2308)	510 ± 274 (93–1243)	0.020
BPE% (%): mean ± SD (range)			
SUB1	45 ± 9 (21–62)	40 ± 11 (18–62)	0.011
SUB2	58 ± 11 (31–78)	51 ± 13 (23–77)	0.006
SUB3	62 ± 11 (36–82)	56 ± 14 (28–80)	0.014

FGT = Fibroglandular tissue.

BPE = Background parenchymal enhancement.

SUB1, SUB2, SUB3 = Subtraction sequence (i.e., post-contrast – pre-contrast) for each of first, second, and third post-contrast sequences, respectively.

*Shown BPE and FGT measure summaries are from the contralateral negative breast of both cancer cases and controls. The p-values are for comparison of controls to cancer cases using paired t-test.

### Associations of BPE measures with breast cancer presence

In this study cohort, none of mammographic density, |FGT|, or FGT% predicted case/control status in univariate or multivariable models (p > 0.4). BPE measured in different SUBs showed an association with breast cancer (Table 3). As seen in Comparison A (using contralateral breast of controls), univariate conditional logistic regression results reflected the odds of malignancy 1.5 times higher per 200 cm^3^ increase in |BPE| and 3.1 times higher per 20 percentage point increase in BPE% (SUB1). Compared to the univariate models, odds ratios for BPE measures were slightly higher in multivariable models controlling for menopausal status, family history of breast cancer, BI-RADS-based mammographic density, |FGT|, and FGT%. We found that the odds ratios were similar for the three subtracted sequences (SUB1, SUB2, and SUB3). After applying multiple test control for 6 comparisons at the 0.05 overall FDR, the 6 adjusted p-values remained statistically significant (all p < 0.031 for the 6 univariate analyses and all p < 0.021 for the 6 multivariable analyses).

**Table 3:** Breast cancer odd ratios for BPE quantified separately from three SUBs for 51 women with a cancer diagnosis and 51 controls with a biopsy-proven benign finding.

		SUB1	SUB2	SUB3
**Comparison A: Contralateral breasts of both cancer cases and controls**				
|BPE|	Univariate	1.5 (1.1, 2.2); p = 0.026	1.4 (1.0, 1.8); p = 0.025	1.3 (1.0, 1.7); p = 0.031
	Multivariable	2.0 (1.1, 3.7); p = 0.021	1.9 (1.1, 3.0); p = 0.014	1.8 (1.1, 2.8); p = 0.017
BPE%	Univariate	3.1 (1.2, 7.9); p = 0.018	2.5 (1.2, 5.3); p = 0.012	2.3 (1.1, 4.5); p = 0.021
	Multivariable	3.5 (1.3, 9.8); p = 0.015	2.9 (1.3, 6.6); p = 0.010	2.5 (1.2, 5.1); p = 0.015
**Comparison B: Benign breast of controls vs contralateral breasts of cancer cases**				
|BPE|	Univariate	1.5 (1.0, 2.1); p = 0.037	1.4 (1.0, 1.8); p = 0.031	1.3 (1.0, 1.7); p = 0.038
	Multivariable	2.3 (1.1, 4.8); p = 0.032	1.9 (1.1, 3.4); p = 0.020	1.8 (1.1, 3.1); p = 0.028
BPE%	Univariate	7.0 (1.6, 30.5); p = 0.010	4.7 (1.6, 13.7); p = 0.005	3.3 (1.2, 9.1); p = 0.023
	Multivariable	7.4 (1.6, 35.6); p = 0.012	5.5 (1.7, 18.4); p = 0.005	3.5 (1.2, 10.0); p = 0.020

|BPE| = Volume of background parenchymal enhancement, with odds ratio for 200 cm^3^ difference.

BPE% = Percentage of background parenchymal enhancement volume (|BPE|) relative to breast volume, with odds ratio for 20 percentage point difference.

SUB1, SUB2, SUB3 = Subtraction sequence (i.e., post-contrast – pre-contrast) for each of first, second, and third post-contrast sequences, respectively.

Odds ratios, 95% confidence intervals, and p-values are shown for univariate conditional logistic regression, and for multivariable models controlling for menopausal status, family history of breast cancer, BI-RADS-based mammographic density, |FGT|, and FGT%. The p-values indicate statistical significance of the tested BPE measure (i.e., |BPE| or BPE%) in the logistic regression modeling. The p-values shown in the table were prior to the multiple test adjustment by FDR. After applying the adjustment for 6 comparisons at the 0.05 overall FDR, the 6 adjusted p-values remained statistically significant (all p < 0.031 for the 6 univariate analyses in Comparison A; all p < 0.021 for the 6 multivariable analyses in Comparison A; all p < 0.038 for the 6 univariate analyses in Comparison B; all p < 0.032 for the 6 multivariable analyses in Comparison B).

In Comparison B (using benign breast of controls), we found overall similar results on the association of BPE with breast cancer (Table 3). We noticed that the odds for BPE% almost doubled the corresponding values in Comparison A. After applying the FDR adjustment, the p-values also remained statistically significant (all p < 0.038 for the 6 univariate analyses and all p < 0.032 for the 6 multivariable analyses).

Similar trends/results were observed for various robustness analyses as well. When excluding the six pairs for whom the cancer cases had additional high-risk factors as described above, odds ratios remained similar. Similar odds ratios were also observed when the 10 mismatched pairs in menopausal status were excluded from analysis. In menopausal status subgroup analysis, the trend still held for the 17 postmenopausal pairs (odds ratio = 13.6, p = 0.048) and the 24 premenopausal pairs (odds ratio = 2.4, p = 0.17), despite the smaller sample size.

### BPE quantified from different SUBs on distinguishing cancer cases from controls

**Figure 2:**
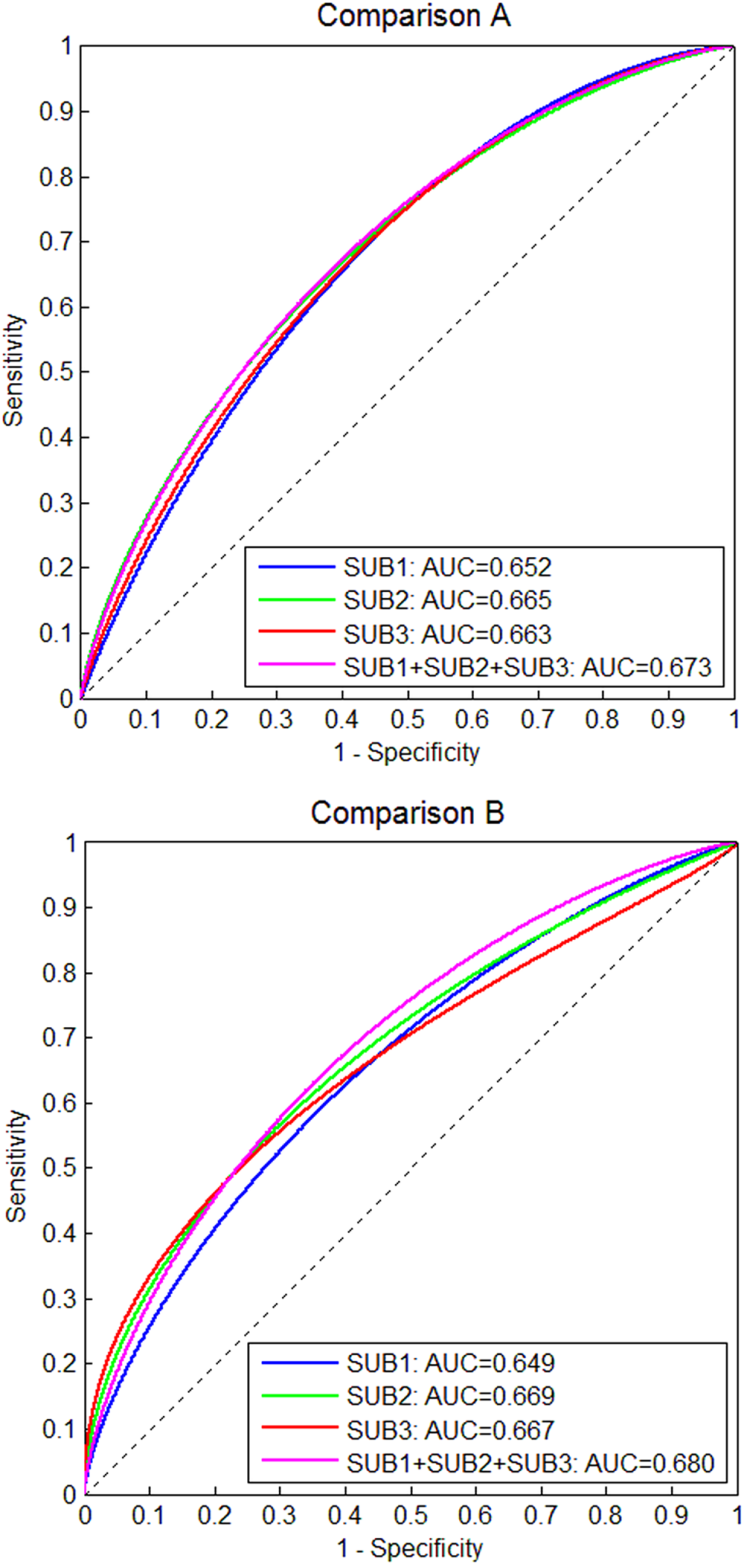
AUCs of the combination of |BPE| and BPE% computed from different SUBs (SUB1, SUB2, and SUB3) in Comparison A (top) and Comparison B (bottom).

The AUCs of the measures of |BPE| and BPE%, individually or combined, for distinguishing cancer cases from controls, are shown in Table 4. AUCs were not significantly different (p > 0.2) for all three SUBs and their combination. The percentage BPE measure (BPE%) showed a greater AUC than the absolute volume measure (|BPE|), consistently in three SUBs, although the difference was not statistically significant (p > 0.05). Combining |BPE| to BPE% generated a non-significant increase in AUC (except for SUB3 where p < 0.05) compared to BPE% alone. The ROC curves of the combination of |BPE| and BPE% computed from the three SUBs were shown in Fig. 2.

**Table 4:** AUCs of the unconditional logistic regression analyses for testing the BPE measures computed from three different SUBs on distinguishing cancer cases from controls.

	SUB1	SUB2	SUB3	SUBs 1, 2, and 3 combined	^*^P-values
**Comparison A: Contralateral breasts of both cancer cases and controls**					
|BPE|	0.612	0.615	0.609	0.612	all p > 0.59
BPE%	0.639	0.654	0.629	0.657	all p > 0.36
|BPE| + BPE%	0.652	0.665	0.663	0.673	all p > 0.41
^+^P-values	all p > 0.29	all p > 0.22	all p > 0.016	all p > 0.12	
**Comparison B: Benign breast of controls vs contralateral breasts of cancer cases**					
|BPE|	0.595	0.606	0.595	0.611	all p > 0.20
BPE%	0.614	0.653	0.624	0.654	all p > 0.25
|BPE| + BPE%	0.649	0.669	0.667	0.680	all p > 0.32
^+^P-values	all p > 0.061	all p > 0.12	all p > 0.0047	all p > 0.11	

|BPE| = Volume of background parenchymal enhancement.

BPE% = Percentage of background parenchymal enhancement volume (|BPE|) relative to breast volume.

SUB1, SUB2, SUB3 = Subtraction sequence (i.e., post-contrast – pre-contrast) for each of first, second, and third post-contrast sequences, respectively.

^*^P-values represent the DeLong’s test between any pair of the AUCs with respect to SUB1, SUB2, SUB3, and their combination (i.e., columns 2–5).

^+^P-values represent the DeLong’s test between any pair of the AUCs with respect to |BPE|, BPE%, and their combination (i.e., rows 3–5 for Comparison A and rows 8–10 for Comparison B).

### BPE versus basic risk factors on distinguishing cancer cases from controls

**Figure 3:**
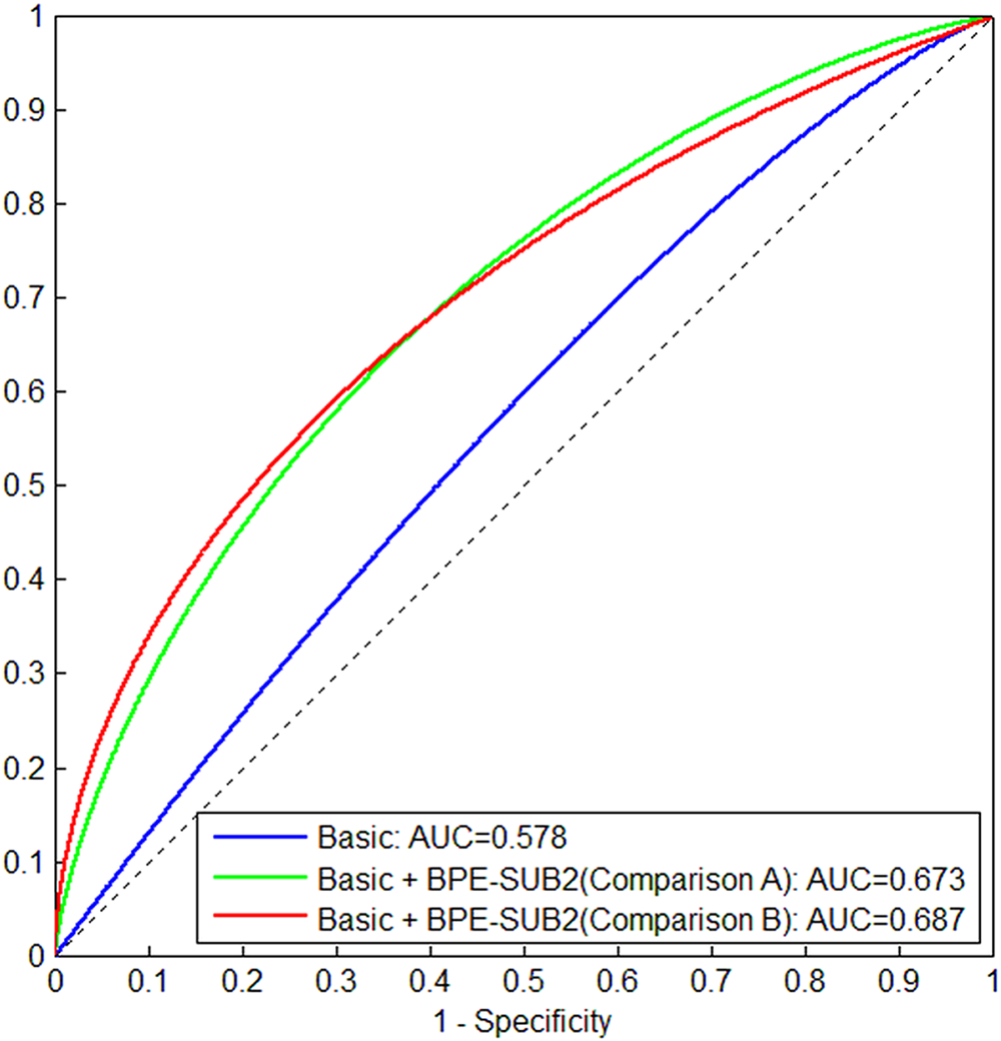
AUCs of distinguishing cancer cases from controls when using only four basic factors (i.e., age, menopausal status, family history, and ordinal mammographic density) and when the combination of |BPE| and BPE% computed from SUB2 were added to the four basic factors.

As shown in Fig. 3, when using only four basic factors (i.e., age, menopausal status, family history, and ordinal mammographic density), the AUC of unconditional logistic regression in distinguishing cancer cases from controls was 0.578. When the combination of |BPE| and BPE% computed from SUB2 (we chose SUB2 because it achieved a greater AUC compared to SUB1 and SUB3) were added to the four basic factors, AUC was 0.673 in Comparison A and 0.687 in Comparison B, respectively, both with a statistically significant increase (p = 0.0029 in Comparison A and p = 0.0026 in Comparison B) relative to the AUC of using the four basic factors alone.

As an exploratory analysis for the 51 cancer cases, we found that none (all p > 0.15, using SCC, Wilcoxon rank-sum test, or Fisher’s exact test as appropriate) of the quantitative FGT, BPE measures (from any of the three post-contrast sequences), and basic risk factors (i.e., age, menopausal status, mammographic density, and family history) was associated with tumor size or tumor type (i.e., tumors involving IDC [n = 45] vs others [DCIS and ILC, n = 6]).

## Discussion

Qualitatively assessed breast MRI BPE using the BI-RADS categories has been studied on its relationship with breast cancer presence or risk^6–8, 23^. Two case-control studies^6, 7^ reported an association between qualitative BPE and breast cancer risk in high-risk screening cohorts. While a recent study^8^ showed an association of BPE with breast cancer presence, another study^23^ on non-high-risk patients found an opposite result. The difference in the nature of the study populations may account for the discrepant findings of these previous studies. In the present work, we performed a quantitative case-control study and showed that fully automated MRI BPE measures (both |BPE| and BPE%) are associated with breast cancer presence. Our findings are in line with several previous qualitative reader studies^6–8^, wherein a variety of odds ratios for BI-RADS-based BPE assessment were reported. Moreover, we showed that BPE measured in three different SUBs has a similar effect on its association with breast cancer presence. Our study therefore added a new contribution to the literature in examining the relationship of BPE measures with breast cancer presence/risk.

In our study cohort, neither BI-RADS-based mammographic density categories nor quantitative MRI FGT measures were correlated with breast cancer presence. In previous reader studies, MRI FGT showed a weak^6^ or null^7^ association with breast cancer risk. Together, it seemed that the association between BPE and breast cancer presence/risk may be independent of mammographic density and MRI FGT, but further validation is warranted for this finding.

Per the American College of Radiology (ACR) guidelines, the first post-contrast sequence is suggested for BI-RADS-based BPE assessment^9^. We quantified BPE levels in each of the three post-contrast sequences of the standard DCE-MRI protocol at our institution. Our results showed that the effects of association between BPE and breast cancer presence were similar for the three post-contrast sequences or SUBs: SUB1 had a slightly larger odds ratio than SUB2 or SUB3, and there is no statistically significant difference in terms of the AUC performance across the three SUBs. These findings imply that for computing quantitative BPE as a potential breast cancer presence/risk biomarker, any of the three post-contrast sequences may be equivalent in the context of breast MRI screening. More specifically, a single early post-contrast sequence (SUB1) may be adequate for use in a practical clinical workflow. This finding may be in line of an emerging abbreviated breast MRI screening protocol—the FAST MRI techniques^16, 17^ —which intended to use only the first post-contrast subtracted sequence (and the Maximum Intensity Projection images) for screening and had shown a similar screening performance with using the full DCE-MRI sequences^17^.

Currently, no standard value has been established for the intensity enhancement ratio threshold R%_cutoff_ in quantifying BPE, as BPE has been assessed qualitatively in the past^6–9, 23^. In computer-aided breast cancer detection/diagnosis software, an R% cutoff threshold value of 30% or 50% was clinically observed^24^. The lower cutoff value of 20% reported here, may reflect specific/different properties associated with characterizing the contrast enhancement on normal breast tissues for studying breast cancer risk. In the robustness analysis of testing BPE measures quantified at R%_cutoff_ = 30% and 40%, we found similar results with R%_cutoff_ = 20%, indicating that our quantitative BPE measures may be a fairly robust biomarker for breast cancer, valid across a range of parameter values (i.e., 20%, 30%, and 40%) for R%_cutoff_. We noticed that the contrast-to-noise ratio of breast MR images is affected by DCE-MRI parameters and type of contrast agents. Therefore, the findings on the R%_cutoff_ parameter for BPE quantification are subject to further investigation.

In this work we performed two comparisons (Comparison A and Comparison B), aimed to test the effects of BPE quantified on a mixture of negative and benign-containing breasts. The associations found in both the two comparisons suggest robustness of BPE, in the sense that ultimately we would expect to derive BPE as an imaging biomarker from a wide range of “normal” or “non-diseased” breast tissue. That being said, it needs to point out that the inclusion of the benign lesion in quantifying BPE in benign-containing breasts may have introduced bias in Comparison B. Currently our algorithms lack the function of segmenting the benign lesions out in the breast, which prevented us from an additional robustness analysis by looking into the effects of BPE quantified from the breasts that have excluded the benign lesions.

The strengths of this study include 1) use of fully-automated computer methods yielding objective and reproducible BPE quantification, 2) use of MRI scans acquired with a fairly consistent imaging protocol, reducing complexity of dealing with varying MR imaging protocols and parameters, and 3) robustness analysis using a mixture of negative and benign-finding breasts. Our study has some limitations. This is a retrospective single-institutional study and our sample size is relatively small. Therefore further evaluation is warranted for assessing the generalizability of our findings on a larger breast MRI dataset (possibly a multi-center study), but this proof-of-concept study will be instrumental in guiding appropriate design of larger retrospective and/or prospective studies. We were not able to do a comparison of BPE between our quantitative assessment and the BI-RADS-based assessment, because the BI-RADS-based assessment of BPE was not yet fully implemented or standardized at our clinics during the time the study cohort had the MRI scans (2009–2011) and, therefore, not available for the majority of the MRI scans for analysis. However, this comparison will be feasible when more recent breast MRI scans are analyzed. In addition, the AUCs of 0.689 for BPE alone and 0.687 when combined with the four basic factors are in line with the reported AUCs (range 0.6–0.75^25, 26^) of existing risk models (such as the Gail model and the Claus model). Of note, as the incremental value of a biomarker to improve breast cancer risk prediction cannot be assessed conclusively in matched case-control studies^27–29^, the increased AUCs of BPE measures relative to the basic risk factors should be interpreted with caution; yet, they are still indicative of the preliminary value of BPE in comparing to existing risk factors. Future larger studies are warranted for a further evaluation of BPE’s prediction capability. Finally, we noticed that a group of premenopausal women had their MRI examinations outside the clinically recommended scanning window (second week of the menstrual cycle), which may have introduced variations in the BPE assessment because BPE levels vary between menstrual cycle weeks^30–32^. However, as indicated in previous work^18, 31^, the measured BPE outside the second week in the control group would have yielded a higher value than the actual levels, attenuating the difference of BPE between cancers and controls in the case-control analysis. Despite this attenuation, we still found a significant difference of the BPE measures between the cancer and control groups on all of the three SUBs.

In summary, this preliminary study showed that fully automated quantitative assessment of breast MRI BPE is associated with breast cancer presence and the association has a similar effect across the three sequential post-contrast DCE sequences. Our study supports further investigation of BPE as a potential biomarker of breast cancer risk, where a single early post-contrast sequence may be adequate for estimating breast cancer risk in breast cancer MRI screening. Quantitative MRI BPE is expected to ultimately improve breast cancer risk prediction^33^ and have direct benefits to enhance clinical utility of screening breast MRIs and to aid in more informed breast cancer risk management^34^.
